# Supplementary material for: Anthocyanin Content and Fusarium Mycotoxins in Pigmented Wheat (Triticum aestivum L. spp. aestivum): An Open Field Evaluation
Source: Plants (Basel). 2023 Feb 4;12(4):693. doi: 10.3390/plants12040693 (PMC9965368; doi:10.3390/plants12040693)
Supplement: Supplementary file 1 [file plants-12-00693-s001.zip › plants-2122234-supplementary.pdf]

Supplementary Files for:

Article

Anthocyanin Content and *Fusarium* Mycotoxins in Pigmented Wheat (*Triticum aestivum* L. spp. *aestivum*): An Open Field Evaluation

Marco Gozzi [1], Massimo Blandino [2], Chiara Dall'Asta [1], Petr Martinek [3], Renato Bruni [1,*] and Laura Righetti [1,*,†,‡]

[1] Department of Food and Drug, University of Parma, Parco Area delle Scienze 27/a, 43100 Parma, Italy; marco.gozzi@unipr.it (M.G.); chiara.dallasta@unipr.it (C.D.)
[2] Department of Agricultural Forest and Food Sciences, University of Turin, Largo Paolo Braccini 2, 10095 Grugliasco, Italy; massimo.blandino@unito.it
[3] Agrotest Fyto, Ltd., Havlíčkova 2787/121, 767 01 Kroměříž, Czech Republic; martinek@vukrom.cz
* Correspondence: renato.bruni@unipr.it (R.B.); laura.righetti@wur.nl (L.R.)
† Present Address: Wageningen Food Safety Research, Wageningen University & Research, P.O. Box 230, 6700 AE Wageningen, The Netherlands. .
‡ Present Address: Laboratory of Organic Chemistry, Wageningen University, 6708 WE Wageningen, The Netherlands.

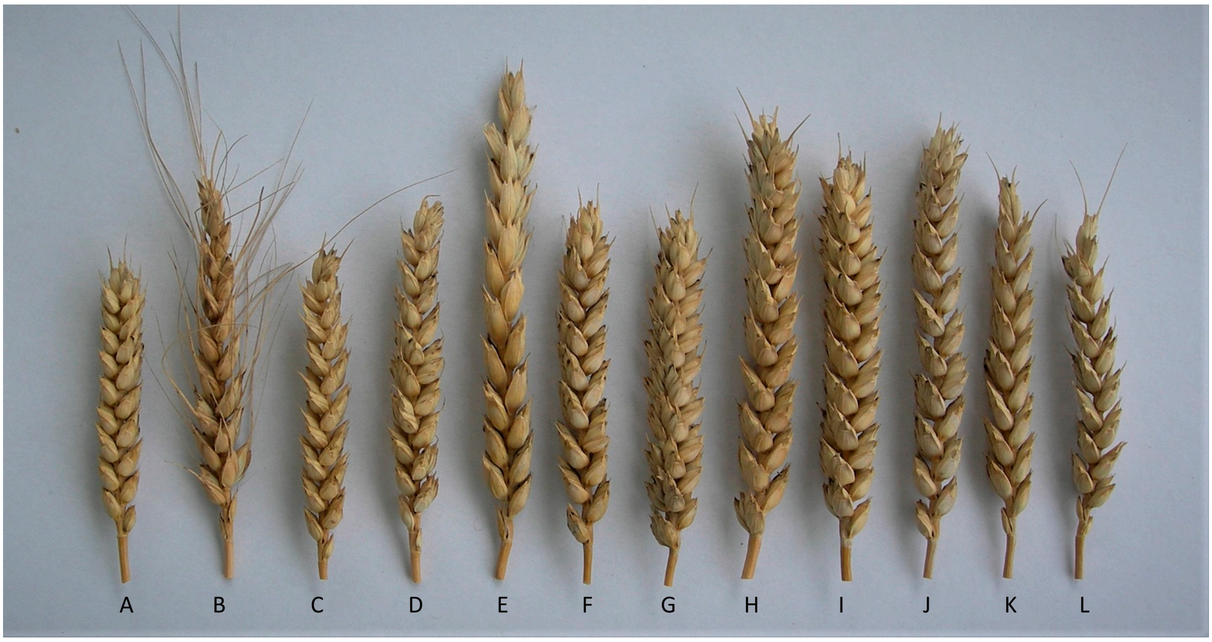

Figure S1 : Appearance of ears of used varieties: A – Aubusson; B – Anthograin; C – Ceraso; D – AF Jumiko; E – KM 106-18 (with elongated glume transferred from Triticum polonicum L.); F – Merlot; G – Rosso; H – Skorpion; I – AF Oxana; J – KM 72-18; K – AF Zora; L – KM 98-18

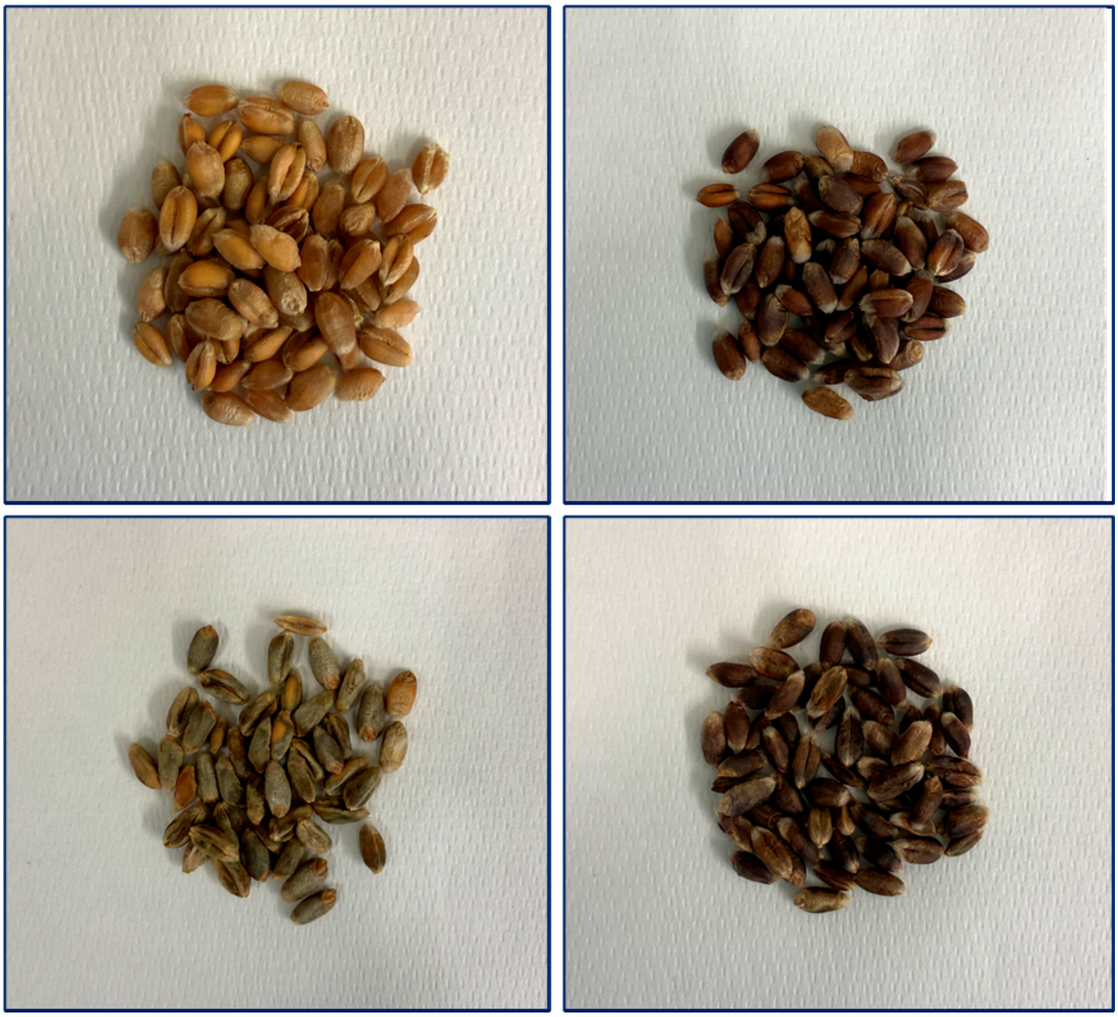

Figure S2. Appearance of grains of different colour selected between the cultivars /breeding lines considered in this study: Aubusson (top left), Merlot (top right), Skorpion (bottom left) and AF Zora (bottom right).
